# Evaluation of Fitness and Accuracy of Milled and Three-Dimensionally Printed Inlays

**DOI:** 10.1055/s-0042-1758796

**Published:** 2023-01-04

**Authors:** Yoen Ah Lim, Jeong Mi Kim, Yoorina Choi, Sujung Park

**Affiliations:** 1 Department of Conservative Dentistry, School of Dentistry, Wonkwang University, Iksan, Republic of Korea; 2 Wonkwang University Dental Hospital, Central Dental Laboratory, Iksan, Republic of Korea

**Keywords:** 3D print, inlay, fitness, precision

## Abstract

**Objective** This article compares and evaluates the marginal and internal fitness and three-dimensional (3D) accuracy of class II inlays fabricated using Tescera (TS) resin, milling of hybrid and zirconia blocks, and 3D printing with NextDent C&B.

**Materials and Methods** Fifty-two mesio-occlusal inlays were fabricated using conventional method with TS, milling of Lava Ultimate (LU), milling of Zolid Fx multilayer (ZR), and 3D printing ( *n* = 13 each). The marginal and internal fitness were evaluated at six points in the mesio-distal section of a replica under a digital microscope (160× magnification), and the accuracy was evaluated using 3D software. Analyses were conducted using *t* -test, one-way analysis of variance (ANOVA) and two-way ANOVA, while Duncan's multiple range test was used for post hoc analyses ( *α* = 0.05).

**Results** The marginal and internal fitness of the 3D and ZR were significantly superior to that of the TS and LU. For LU, ZR, and 3D, a significant discrepancy between the marginal gap and internal gap was observed ( *p* < 0.05). On evaluating accuracy, trueness was significantly higher in ZR than in TS and LU; precision was significantly higher in 3D and ZR than in TS and LU ( *p* < 0.05).

**Conclusion** The marginal and internal fitness and the accuracy of TS, ZR, and 3D were within the clinically acceptable range. The marginal and internal fitness and accuracy of 3D were better than those of TS and LU, which are commonly used in dentistry. There is immense potential for using 3D-printed inlays in routine clinical practice.

## Introduction

Computer-aided design/computer-aided manufacturing (CAD/CAM) was introduced in dentistry in the 1980s and has an extensive range of applications. 1 It is divided into subtractive (milling) and additive (three-dimensional [3D] printing and rapid prototyping) methods depending on the process of fabrication. 2 3 Compared with additive method, the disadvantages of subtractive method include wastage of material, frequent replacement of equipment due to wear, and higher possibility of defects due to poor machinability when working on high-strength blocks. 1 3 4 Contrarily, the additive method has no limitations on the output size, can be used to fabricate complicated designs, and causes no material waste. Hence, it is preferred in dentistry. 5

Among the 3D printing methods, selective laser sintering, selective laser melting, stereolithography (SLT), and digital light processing (DLP) are commonly used in dentistry. 6 SLT and DLP are more frequently used due to small equipment size, excellent cost-effectiveness, and ability to print high-resolution, sophisticated outputs; among these, DLP is more popular due to its faster printing speed than SLT. 1 3 5

Marginal gap (MG) and internal gap (IG) are critical factors affecting the long-term success of dental prostheses. Larger MG increases the risk of leakage from dissolution and wear of cement caused by physical fatigue and chemical corrosion; the resultant subgingival plaque and bacterial infiltration may lead to hypersensitivity, secondary caries, and prosthesis failure. Poor internal fitness leads to decreased retention and support of the prosthesis and increased cement thickness, thereby lowering the fracture resistance of the prosthesis. 7 8

Although difficult to obtain in clinical practice, the American Dental Association Specification No. 8 stipulates that a gap of 25 to 40 µm is appropriate for optimal fitness of fixed prostheses; however, McLean and von Fraunhofer suggested a clinically acceptable limit of 120 µm. 9 Many studies have reported that an MG and IG of < 120 µm is clinically satisfactory. 10 The accuracy of 3D-printed prostheses has been reported by various studies to be within the clinically acceptable range compared with those made by milling or the traditional lost wax technique. 3 4 One study suggested an average MG of 50 to 60 and 150 to 168 µm in ceramic crowns and mesio-occluso-distal ceramic inlays fabricated by CAD/CAM 11 ; another study reported that the MG increased with more complex cavity geometry, such as with cusp capping. 12

Nondestructive methods for evaluating MG and IG include micro-computed tomography and the replica technique. 13 Of these, the replica technique has some disadvantages, including difficulties in identifying the margins, potential errors when the silicone impression is torn or cut, and a limited number of measurement points 14 15 16 17 ; additionally, the accuracy may be affected by the type of silicone material, measurement method, and number of measurement points used. 18 Nevertheless, the replica technique is generally accepted as an easy, reliable, and less expensive measurement method for MG and IG. 17 19 20

For evaluating accuracy, the trueness and precision of the measured data were evaluated against the reference data obtained from 3D scanning in accordance with the International Organization for Standardization 5725–1 standard, and the results were expressed as root mean square (RMS). Lower RMS values indicate superior trueness and precision. Previous studies have suggested that RMS values < 10 µm indicate excellent fitness, whereas RMS values > 50 µm indicate poor fitness. 21 22 Although trueness does not measure the fitness, it is used to evaluate the clinical quality by comparing the difference between reference data and measured data from the fabricated prosthesis. 23 The measured data may be affected by scanner accuracy and data analysis method; however, 3D accuracy is reported to be a more reliable method than two-dimensional evaluation since a computer could be used to measure the 3D distance from the measurement points and additional random points. 21

With an increase in the use of 3D printing in dentistry, many studies have investigated the MG and IG or accuracy of 3D-printed provisional crowns, orthodontic models, abutment models, and full dentures. Nonetheless, evidence is scarce on the fitness and accuracy of 3D-printed resin inlays.

In this study, we compared class II inlays made by conventional method using Tescera resin (TS), milling Lava Ultimate (LU), and Zolid Fx multilayer (ZR) blocks, which are widely used in clinical practice for esthetic restoration, and 3D printing using NextDent C&B resin. The null hypotheses tested were: (1) there is no difference in the marginal and internal fitness and accuracy according to the material, and (2) there is no difference according to the measurement point between IG and MG in each group.

## Materials and Methods

**Fig. 1:**
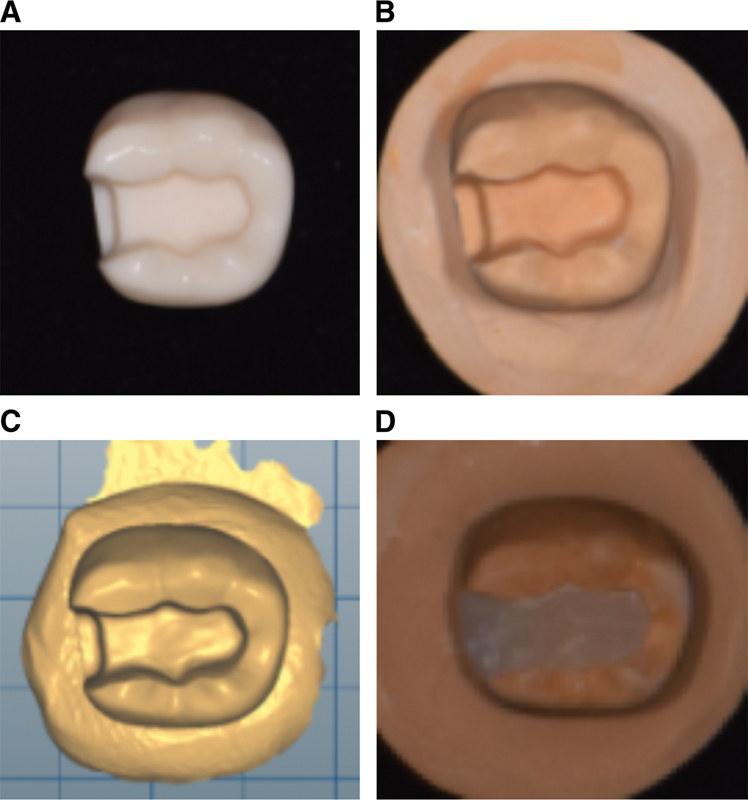
Inlay cavity preparation and fabricated inlay specimen. ( **A** ) Mandibular right first molar with a prepared class II mesio-occlusal inlay cavity, ( **B** ) master die, ( **C** ) image of the master die saved as standard tessellation language file of master die, and ( **D** ) inlay specimen fabricated by 3D printing. 3D, three-dimensional.

A mesial class II inlay cavity (occlusal box depth = 2.5 mm and isthmus width = 3.0 mm; proximal box with mesiodistal width = 1.2 mm, buccolingual width = 3.0 mm, and occlusogingival depth = 4.0 mm; and convergence angle = 8 degrees) was prepared on a mandibular right first molar resin tooth (Nissin Dental Products, Kyoto, Japan) using a high-speed handpiece with a diamond burr (TF-31, MANI, Tochigi, Japan). Silicon impression material (Honigum Light Body, Regular Body, DMG, Hamburg, Germany) was used to make an impression of the prepared cavity. Using high-strength dental stone (Fujirock EP, GC Europe), 52 master dies ( Fig. 1 ) were made. Subsequently, 52 class II inlays were fabricated using the four different materials in accordance with the manufacturer's protocol. The experimental groups were conventional preparation using TS, milling of LU, milling of ZR, and 3D printing with NextDent C&B resin ( Table 1 ). The number of inlay specimens in each group was 13.

**Table 1:** Materials assessed

Group	Brand name	Manufacturer	Composition		
			Monomer	Filler (mass%)	
TS	Tescera	Bisco, Chicago, IL, USA	Ethoxylated bis-GMA, UDMA	Glass frit amorphous silica	< 80 < 25
LU	Lava Ultimate	3M ESPE, St. Paul, MN, USA	Bis-GMA, UDMA, Bis-EMA, TEGDMA	SiO2 (20 nm), ZrO2 (4–11 nm), aggregated ZrO2/SiO2 cluster (SiO2 = 20 nm, ZrO2 = 4–11 nm)	80
ZR	Zolid Fx multilayer	Amann Girrbach, Pforzheim, Germany		ZrO2 + HfO2 + Y2O3 Y2O3 HfO2 Al2O3 Other oxides	≥ 99.0 8.5–9.5 ≤ 5 ≤ 0.5 ≤ 1
3D	NextDent C&B	3D Systems, Soesterberg, Netherlands	Methacrylic oligomers, phosphine oxides		

Abbreviations: 3D, three-dimensional printing; Bis-EMA, ethoxylated bisphenol-A dimethacrylate; Bis-GMA, bisphenol A diglycidylether methacrylate; LU, milling with Lava Ultimate; TEGDMA, triethylene glycol dimethacrylate; TS, Tescera resin; UDMA, urethane dimethacrylate; ZR, milling with Zolid Fx multilayer blocks.

The TS group specimens were prepared as follows: a resin separator (TS die separator, Bisco, Chicago, Illinois, United States) was applied to the cavity in the master die. After drying, TS resin (Bisco) was placed directly inside the cavity to prepare the external morphology of the inlay, followed by light curing for 2 minutes in a TS ATL Light Cup (Bisco). After adding enough water to submerge the specimen and one spoon of dissolved oxygen scavenger (TS Oxygen Scavenger Plus, Bisco) to the TS ATL Heat Cup, the specimen was placed and heat-cured for 25 minutes at 130°C in accordance with the manufacturer's protocol.

In the LU, ZR, and 3D groups, class II inlay specimens were prepared as follows: after scanning the master die using an intraoral scanner (Trios 3, 3shape, Copenhagen, Denmark), the Ceramill Mind (Amann Girrbach, Pforzheim, Germany) program was used to design the class II inlay specimens with 30 µm of cement space 1.0 mm above the margin. The specimens were designed and fabricated according to each manufacturer's protocol.

In the LU and ZR groups, CAD/CAM milling equipment (Ceramill Motion 2, Amann Girrbach) was used to mill the hybrid blocks (LU, 3M ESPE, St. Paul, Minnesota, United States) and zirconia blocks (ZR multilayer, Amann Girrbach). The milled ZR specimens were placed in a heating furnace (Ceramill Therm, Amannn Girrbach), and the temperature was increased from 20°C to 1,450°C at a rate of 12°C/min. Subsequently, the specimens were left at 1,450°C for 1 hour before being cooled.

In the 3D-printed group, NextDent C&B resin (3D Systems, Soesterberg, Netherlands) and a DLP 3D printer (Bio3D L12 Dental Professional, BIO3D, Seoul, Republic of Korea) were used to print the specimens with a layer thickness of 50 µm, printing rate of 10 mm/h, and building angle of 180 degrees. Subsequently, the specimens were placed inside a light-curing unit (LC-3Dprint Box, 3D Systems) and cured for 30 minutes.

**Fig. 2:**
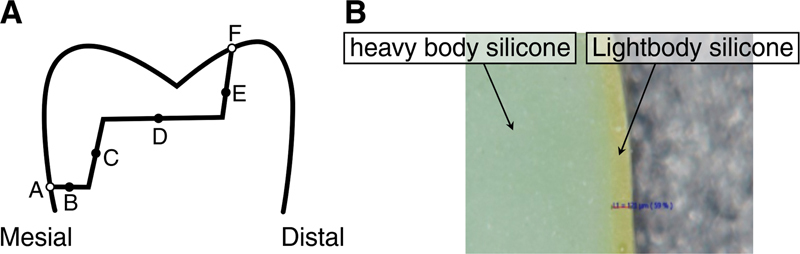
( **A** ) Schematic representation of measurement points for the marginal and internal discrepancy in the sagittal section of silicone replica: marginal areas = A, F; internal areas = B, C, D, E. ( **B** ) Measurement of the internal gap of Zolid Fx multilayer by digital microscope at 160× magnification (light green: light body silicone, green: heavy body silicone).

The replica for the evaluation of marginal and internal fitness was prepared as follows: after injecting light body silicone (Aquasil Ultra XLV, Dentsply DeTrey, Konstanz, Germany) into the cavity of the master die ( Fig. 1A ) and inserting the inlay specimen within its position in the cavity, a universal testing machine (Instron 3345, Instron Corporation, Norwood, Massachusetts, United States) was used to apply a load of 5 N for 5 minutes until the silicone hardened. 24 On the mesiodistal portion of the replica that passed through the central fossa, the MG was measured at two points, cervical margin (A) and occlusal margin (F), and the IG was measured at four points, the midpoints of cervical floor (B), axial wall (C), pulpal wall of the occlusal box (D), and axial wall of occlusal box (E), under a digital microscope (KH-7700, Hirox, Tokyo, Japan) at 160× magnification ( Fig. 2 ).

After evaluating marginal and internal fitness, accuracy was assessed. The standard tessellation language (STL) files obtained by scanning the inlay cavity of the master die were used as the reference data for the TS group, while STL files designed by CAD software were used as reference data for the other three groups. Measurement data were generated as STL files by scanning the prepared inlay specimens with a model scanner (Identica Blue, Medit, Seoul, Republic of Korea). The generated data files were evaluated for trueness and precision using 3D software (Geomagic Verify, 3D Systems Inc., United States). Trueness was evaluated by comparing the discrepancy between the reference and measurement data files in each experimental group ( *n* = 13). Precision was calculated by comparing pairs of measurement data files in each experimental group ( *n* = 78).

To determine the deviation between the reference and measurement data files, two data files were superimposed to measure trueness and precision. The RMS value was used to quantify the trueness and precision, and was calculated as follows:



where *x*_1_ is the reference data at measurement point *i* , *x*_2_ is the measurement data at measurement point *i* , and *n* is the total number of measurement points. A smaller RMS value indicated a higher accuracy.

For visualizing the differences between the reference and measurement data, 3D deviation image analysis was used to obtain a 3D color map. Areas where the measured data differed within ± 10 µm from the reference data were displayed in green, areas where the measured data differed greater than +10 µm were displayed in yellow to red, and areas smaller than –10 µm were displayed in blue to dark blue.

All data were evaluated using SPSS ver. 20.0 statistical software (IBM Corp., Armonk, New York, United States). The interaction effect between the material (TS, LU, ZR, and 3D) and measurement point (MG and IG) was analyzed using two-way analysis of variance (ANOVA). The difference in marginal and internal fitness according to material used was analyzed using one-way ANOVA. The discrepancy between MG and IG in each experimental group was analyzed using *t* -test ( *α* = 0.05). Trueness and precision, representing accuracy, were analyzed by one-way ANOVA, and post hoc analyses were performed using Duncan's new multiple range test ( *α* = 0.05).

## Results

Table 2 shows the results obtained in the evaluation of marginal and internal fitness in the four experimental groups. On comparing MG among the four experimental groups (mean of the values at A and F), the MG of ZR and 3D was statistically significantly smaller than that of TS and LU ( *p* < 0.05). All groups were considered clinically acceptable because MG of all four groups was < 120 μm.

**Table 2:** Discrepancy in each group at the measurement points

Measurement point	Material			
	TS	LU	ZR	3D
MG	107.65 ± 67.37 ^a^	118.54 ± 45.54 ^a^	58.35 ± 14.88 ^b^	53.77 ± 16.29 ^b^
IG	103.1 9 ± 43.80 ^a^	168.81 ± 42.67 ^b^	95.69 ± 13.34 ^a^	82.02 ± 8.32 ^a^
Total sum value	104.68 ± 48.50 ^b^	152.05 ± 36.96 ^c^	83.24 ± 11.78 ^a^	72.60 ± 7.29 ^a^

Abbreviations: 3D, three-dimensional printing; IG, internal gap; LU, Lava Ultimate; MG, marginal gap; TS, Tescera; ZR, zirconia.

Note: Values are presented as mean ± standard deviation; unit, µm. Within the same row, the same lowercase letters indicate no statistically significant difference in one-way analysis of variance (ANOVA) at *α* = 0.05.

On comparing the IG between the groups (mean of the values at B, C, D, and E), the IG of LU (168.81 ± 42.67) was statistically significantly larger than that of TS, ZR, and 3D and larger than the clinically acceptable range ( *p* < 0.05). On comparing the mean MG and IG, the values were statistically significantly smaller in ZR and 3D than in TS and LU ( *p* < 0.05).

**Fig. 3:**
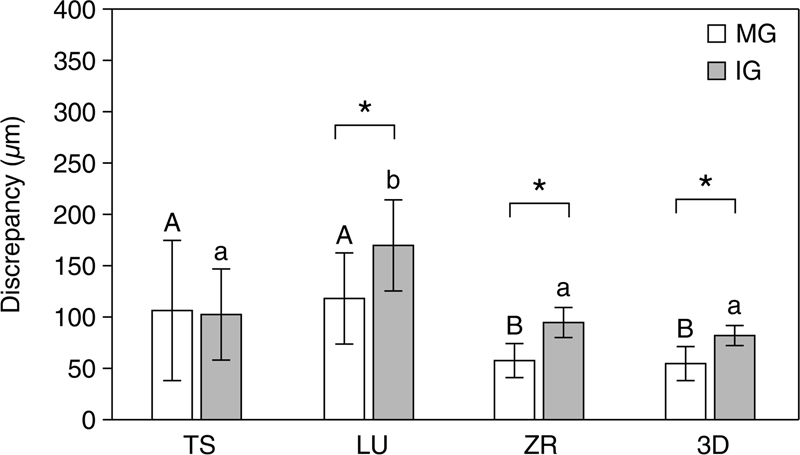
Marginal and internal discrepancies at each measurement point according to materials. The same letters indicate no statistically significant difference by one-way analysis of variance (ANOVA) at *α* = 0.05. *Statistically significant difference by *t* -test at *α* = 0.05. 3D, three-dimensional printing; IG, internal gap; LU, Lava Ultimate; MG, marginal gap; TS, Tescera; ZR, zirconia.

On evaluating the discrepancy between MG and IG in each experimental group ( Table 2 ), there was a statistically significant discrepancy between MG and IG in LU, ZR, and 3D ( *p* < 0.05; Fig. 3 ).

A two-way ANOVA showed no interaction effect between material and measurement point ( *p* > 0.05). There was a difference in the effect of materials and measurement points on fitness, as explained by 47.8%. The material (7.93%) was a more important factor in determining fitness than the measurement point (35.66%) ( Table 3 ).

**Table 3:** Two-way ANOVA considering two factors with the interaction term

Source	Sum of squares	df	Mean squares	*F*	*p*
Corrected model	121461.523	7	17351.646	12.550	0.000
Material	90653.483	3	30217.828	21.857	0.000
Measurement point	20167.578	1	20167.578	14.587	0.000
Material a (measurement point)	10640.463	3	3546.821	2.565	0.059
Error	132725.038	96	1382.552		
Corrected total	254186.562	103			

Abbreviations: ANOVA, analysis of variance; df, degrees of freedom.

Note: *R* ^2^ = 0.478.

a Significantly different by two-way ANOVA at *α* = 0.05.

Regarding trueness, a factor of accuracy, the RMS value of ZR was statistically significantly lower than that of TS and LU ( *p* < 0.05), while no significant difference was identified in the trueness of 3D compared with that of TS, LU, and ZR ( *p* > 0.05; Table 4 ).

**Table 4:** Trueness (RMS values) of the inner surface of the inlays made of four fabrication methods

Group	Trueness (RMS ± SD)	*F*	*p*
TS	47.06 ± 14.73 ^b^	4.360	0.009
LU	48.97 ± 17.49 ^b^		
ZR	33.26 ± 7.24 ^a^		
3D	40.86 ± 4.78 ^ab^		

Abbreviations: 3D, three-dimensional printing; LU, Lava Ultimate; RMS, root mean square; SD, standard deviation; TS, Tescera; ZR, zirconia.

Note: Unit, µm. The same lowercase letters were not significantly different by one-way analysis of variance (ANOVA) at *α* = 0.05.

**Fig. 4:**
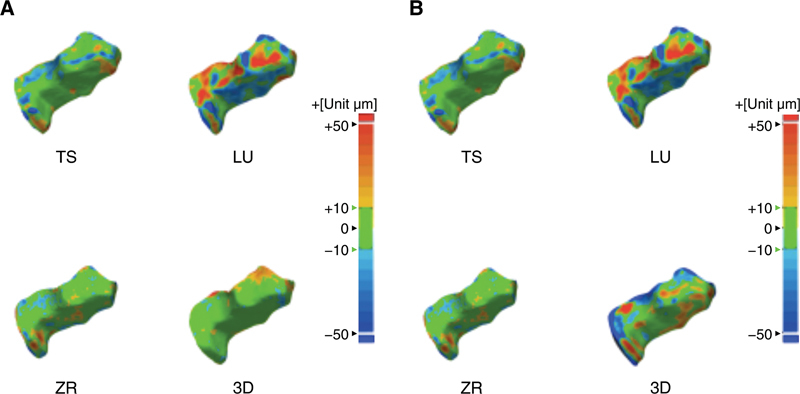
The three-dimensional color deviation maps for trueness and precision of four groups. Positive deviation is displayed as yellow to red colors and negative deviation as blue to dark blue. 3D, three-dimensional printing; LU, Lava Ultimate; TS, Tescera; ZR, zirconia.

The 3D color deviation maps in Fig. 4 show the 3D surface deviation between the reference and measurement data. Yellow to red areas indicate positive deviation and blue to dark blue areas indicate negative deviation. The 3D color deviation map of trueness and precision of LU showed more positive and negative deviations than the other experimental groups.

On evaluating precision, another component of accuracy, the RMS value of 3D and ZR was statistically significantly lower than that of TS and LU, while the RMS value of LU (65.08 ± 13.34) was larger than the clinically acceptable range and showed a significant difference compared with the other three groups ( *p* < 0.05; Table 5 ).

**Table 5:** Precision (RMS values) of the inner surface of the inlays made using the four fabrication methods

Group	Precision (RMS ± SD)	*F*	*p*
TS	45.72 ± 5.68 ^b^	101.698	0.000
LU	65.08 ± 13.34 ^c^		
ZR	24.66 ± 2.35 ^a^		
3D	20.05 ± 2.01 ^a^		

Abbreviations: 3D, three-dimensional printing; LU, Lava Ultimate; RMS, root mean square; SD, standard deviation; TS, Tescera; ZR, zirconia.

Note: Unit, µm. The same lowercase letters indicate no statistically significant difference by one-way analysis of variance (ANOVA) at *α* = 0.05.

## Discussion

This study evaluated the marginal and internal fitness and accuracy of class II inlays fabricated by conventional method using TS, milling of LU and ZR, and 3D printing with NextDent C&B resin. Differences in marginal and internal fitness and accuracy were identified among the four experimental groups ( *p* < 0.05). ZR and 3D showed significantly better marginal and internal fitness and precision than TS and LU ( *p* < 0.05); additionally, ZR showed better trueness than the other three groups. With TS, ZR, and 3D, the marginal and internal fitness was < 120 µm and the RMS values of trueness and precision were < 50 µm; hence, these three groups were within the clinically acceptable. 23 Therefore, the null hypotheses that there would be no differences in marginal and internal fitness and accuracy according to the material between the four groups and that there would be no differences between IG and MG in each group were rejected.

LU was introduced as a material that causes less wear of the opposing dentition, less susceptibility to fracture, better machinability, and easier repair than other porcelain materials used for esthetic restorations. 20 However, a study compared the internal fitness of inlays fabricated using four different blocks reported that LU had a mean IG of 186 µm, which was larger than that of Vita Enamic and IPS e.max ( *p* < 0.05). That is why LU had lower hardness and flexural strength than the other two ceramic blocks, as such a greater amount of material was removed during milling, leading to its poor fitness. 20

In this study, the LU group showed the poorest marginal and internal fitness and accuracy among all groups. Although the MG of the LU group was within the clinically acceptable range, the IG and RMS value of precision were greater than the clinically acceptable range.

Although the LU and ZR specimens were fabricated by a subtractive method using the same milling machine in this study, ZR showed significantly better marginal and internal fitness and accuracy than TS and LU ( *p* < 0.05), with the lowest RMS value of trueness among all four experimental groups. Zirconia materials were designed and milled with 25% extra size to compensate for the 20 to 25% shrinkage after sintering, 2 25 thereby having better fitness than other milled materials. 7 Zirconia blocks were generally overmilled compared with the reference design, which could be helpful with the insufficient compensation of shrinkage. 23 And the pre-sintering density of zirconia is 40% of the final density, 26 which is softer than the LU block. Furthermore, latest CAD software programs were reportedly able to accurately compensate for shrinkage of the block during sintering. 2

Factors that may affect the fitness of the prosthesis fabricated by milling included the material of the block, milling equipment, milling burr size, and degree of wear of the burr. 8 20 27 Spitznagel et al mentioned that the power and torque of the milling device should be modified according to the type of block during milling. 28 Many dental technicians fabricate dozens of crowns without replacing the burr because there are no definitive manufacturer guidelines about changing the milling tool except following tool breakdown and while milling different materials; the prosthesis fabricated without burr replacement is also considered clinically acceptable. 21 In this study, milling of LU blocks and ZR blocks was performed without burr replacement.

When designing the STL files for ZR, LU, and 3D with the CAD software, a cement space of 30 µm at 1 mm above the margin was provided as recommended by the manufacturer. However, for TS, the scanned data of the master die was converted to the STL files and used as the reference data, and the specimen was fabricated without cement space.

In this study, unlike the other three experimental groups, no cement space was provided for TS, nevertheless it showed larger MG and IG than 3D and ZR ( *p* < 0.05). In TS, the RMS value of trueness was also larger than that of ZR, while precision was significantly larger than that of ZR and 3D ( *p* < 0.05). TS may have shown poorer marginal and internal fitness and accuracy than ZR and 3D, fabricated by the CAD/CAM method, due to the accumulation of errors during wax blockout and manual fabrication of the specimens. 29

On comparing MG and IG in the four experimental groups, three of the experimental groups, except TS in which fabrication was performed without cement space, showed a statistically significant discrepancy between MG and IG ( *p* < 0.05).

In 3D, the MG and IG were the smallest among all four experimental groups and significantly smaller than that of LU and TS ( *p* < 0.05). The precision of 3D was statistically significantly better than that of LU and TS, with the smallest RMS value ( *p* < 0.05). The present study supports previous studies that reported better internal fitness of 3D-printed restorations than milled from resin blocks. 3 30 31 The excellent marginal and internal fitness may have been because the specimens were designed and printed to a larger size to compensate for resin polymerization shrinkage. The excellent precision may have been due to fewer errors among the specimens which were 3D printed simultaneously.

Flexural strengths of TS resin, Synfony resin, and LU block, which are commonly used in clinical practice for aesthetic inlays, were 84.6, 76.8, and 170.5 MPa, respectively. 32 33 Although the flexural strength of NextDent C&B (114–119 MPa, 2.3–2.4 GPa, respectively) specimens reported by Keßler et al was lower than that of LU block, it was higher than that of TS or Synfony resin. 5 We expect to improve not only the flexural strength but also other physical property of 3D-printed materials.

This study had several limitations while evaluating the marginal and internal fitness and accuracy of each experimental group. The same burr was repeatedly used while milling ZR and LU specimens. Additionally, the TS specimens were fabricated with no cement space. Although resins for 3D printing have been approved for midterm use for up to 2 years owing to physical properties, 34 additional long-term studies on the properties of 3D-printed resin are needed to implement the use of 3D-printed inlays for permanent restoration.

## Conclusion

Within the limitations of this current study, it was concluded that:

1. The MG of all experimental groups was within the clinically acceptable range (< 120 µm). The trueness and precision RMS value of the ZR, 3D, and TS groups was within the clinically acceptable range < 50 µm.
2. The marginal and internal fitness and accuracy of 3D-printed resin inlays were within the clinically acceptable range and those of 3D-printed inlays were statistically significantly better than those of inlays fabricated by TS and LU, which are widely used in dentistry; 3D-printed resin inlays have high potential for use in routine clinical practice for esthetic restoration.

## References

[1] Reymus M Fabritius R Keßler A Hickel R Edelhoff D Stawarczyk B Fracture load of 3D-printed fixed dental prostheses compared with milled and conventionally fabricated ones: the impact of resin material, build direction, post-curing, and artificial aging-an in vitro study Clin Oral Investig 2020 24 02 701 710 10.1007/s00784-019-02952-7 31127429

[2] Bousnaki M Chatziparaskeva M Bakopoulou A Pissiotis A Koidis P Variables affecting the fit of zirconia fixed partial dentures: a systematic review J Prosthet Dent 2020 123 05 686 6.92E10 31703922 10.1016/j.prosdent.2019.06.019

[3] Lee W S Lee D H Lee K B Evaluation of internal fit of interim crown fabricated with CAD/CAM milling and 3D printing system J Adv Prosthodont 2017 9 04 265 270 28874993 10.4047/jap.2017.9.4.265 PMC5582092

[4] Patzelt S B Bishti S Stampf S Att W Accuracy of computer-aided design/computer-aided manufacturing-generated dental casts based on intraoral scanner data J Am Dent Assoc 2014 145 11 1133 1140 25359645 10.14219/jada.2014.87

[5] Keßler A Hickel R Ilie N In vitro investigation of the influence of printing direction on the flexural strength, flexural modulus and fractographic analysis of 3D-printed temporary materials Dent Mater J 2021 40 03 641 649 33456026 10.4012/dmj.2020-147

[6] Park S M Park J M Kim S K Heo S J Koak J Y Flexural strength of 3D-printing resin materials for provisional fixed dental prostheses Materials (Basel) 2020 13 18 3970 32911702 10.3390/ma13183970 PMC7559938

[7] Cunali R S Saab R C Correr G M Marginal and internal adaptation of zirconia crowns: a comparative study of assessment Braz Dent J 2017 28 04 467 473 29160399 10.1590/0103-6440201601531

[8] Goujat A Abouelleil H Colon P Marginal and internal fit of CAD-CAM inlay/onlay restorations: a systematic review of in vitro studies J Prosthet Dent 2019 121 04 590 597000 30509548 10.1016/j.prosdent.2018.06.006

[9] McLean J W von Fraunhofer J A The estimation of cement film thickness by an in vivo technique Br Dent J 1971 131 03 107 111 5283545 10.1038/sj.bdj.4802708

[10] Kang S Y Lee H N Kim J H Kim W C Evaluation of marginal discrepancy of pressable ceramic veneer fabricated using CAD/CAM system: additive and subtractive manufacturing J Adv Prosthodont 2018 10 05 347 353 30370025 10.4047/jap.2018.10.5.347 PMC6202431

[11] Reich S Gozdowski S Trentzsch L Frankenberger R Lohbauer U Marginal fit of heat-pressed vs. CAD/CAM processed all-ceramic onlays using a milling unit prototype Oper Dent 2008 33 06 644 650 19051857 10.2341/07-162

[12] Park S H Yoo Y J Shin Y J Cho B H Baek S H Marginal and internal fit of nano-composite CAD/CAM restorations Restor Dent Endod 2016 41 01 37 43 26877989 10.5395/rde.2016.41.1.37 PMC4751205

[13] Alajaji N K Bardwell D Finkelman M Ali A Micro-CT evaluation of ceramic inlays: comparison of the marginal and internal fit of five and three axis cam systems with a heat press technique J Esthet Restor Dent 2017 29 01 49 58 27680508 10.1111/jerd.12271

[14] Homsy F R Özcan M Khoury M Majzoub Z AK Comparison of fit accuracy of pressed lithium disilicate inlays fabricated from wax or resin patterns with conventional and CAD-CAM technologies J Prosthet Dent 2018 120 04 530 536 30318049 10.1016/j.prosdent.2018.04.006

[15] Paul N Raghavendra Swamy K N Dhakshaini M R Sowmya S Ravi M B Marginal and internal fit evaluation of conventional metal-ceramic versus zirconia CAD/CAM crowns J Clin Exp Dent 2020 12 01 e31 e37 31976041 10.4317/jced.55946 PMC6969964

[16] Wu J Xie H Sadr A Chung K H Evaluation of internal fit and marginal adaptation of provisional crowns fabricated with three different techniques Sensors (Basel) 2021 21 03 740 33499198 10.3390/s21030740 PMC7865833

[17] Laurent M Scheer P Dejou J Laborde G Clinical evaluation of the marginal fit of cast crowns–validation of the silicone replica method J Oral Rehabil 2008 35 02 116 122 18197844 10.1111/j.1365-2842.2003.01203.x

[18] Al Hamad K Q Al Quran F A AlJalam S A Baba N Z Comparison of the accuracy of fit of metal, zirconia, and lithium disilicate crowns made from different manufacturing techniques J Prosthodont 2019 28 05 497 503 30719780 10.1111/jopr.13029

[19] Svanborg P A systematic review on the accuracy of zirconia crowns and fixed dental prostheses Biomater Investig Dent 2020 7 01 9 15 10.1080/26415275.2019.1708202 PMC6968690 32010901

[20] Goujat A Abouelleil H Colon P Mechanical properties and internal fit of 4 CAD-CAM block materials J Prosthet Dent 2018 119 03 384 389 28552287 10.1016/j.prosdent.2017.03.001

[21] Cho J H Yoon H I Han J S Kim D J Trueness of the inner surface of monolithic crowns fabricated by milling of a fully sintered (Y, Nb)-TZP block in chairside CAD-CAM system for single-visit dentistry Materials (Basel) 2019 12 19 3253 31590370 10.3390/ma12193253 PMC6803933

[22] Peters M C Delong R Pintado M R Pallesen U Qvist V Douglas W H Comparison of two measurement techniques for clinical wear J Dent 1999 27 07 479 485 10507203 10.1016/s0300-5712(99)00027-5

[23] Al Hamad K Q Al-Rashdan R B Al-Rashdan B A Baba N Z Effect of milling protocols on trueness and precision of ceramic crowns J Prosthodont 2021 30 02 171 176 10.1111/jopr.13245 32856358

[24] Kim K B Kim J H Kim W C Kim H Y Kim J H Evaluation of the marginal and internal gap of metal-ceramic crown fabricated with a selective laser sintering technology: two- and three-dimensional replica techniques J Adv Prosthodont 2013 5 02 179 186 23755345 10.4047/jap.2013.5.2.179 PMC3675292

[25] Denkena B Breidenstein B Busemann S Lehr C M Impact of hard machining on zirconia based ceramics for dental applications Procedia CIRP 2017 65 248 252

[26] Denry I Kelly J R State of the art of zirconia for dental applications Dent Mater 2008 24 03 299 307 17659331 10.1016/j.dental.2007.05.007

[27] Bayrak A Akat B Ocak M Kιlιçarslan M A Özcan M Micro-computed tomography analysis of fit of ceramic inlays produced with different CAD software programs Eur J Prosthodont Restor Dent 2021 29 03 29 10.1922/EJPRD_2046Bayrak06 33393742

[28] Spitznagel F A Boldt J Gierthmuehlen P C CAD/CAM ceramic restorative materials for natural teeth J Dent Res 2018 97 10 1082 1091 29906206 10.1177/0022034518779759

[29] Türk A G Sabuncu M Ünal S Önal B Ulusoy M Comparison of the marginal adaptation of direct and indirect composite inlay restorations with optical coherence tomography J Appl Oral Sci 2016 24 04 383 390 27556210 10.1590/1678-775720160012 PMC4990368

[30] Alharbi N Alharbi S Cuijpers V MJI Osman R B Wismeijer D Three-dimensional evaluation of marginal and internal fit of 3D-printed interim restorations fabricated on different finish line designs J Prosthodont Res 2018 62 02 218 226 29032176 10.1016/j.jpor.2017.09.002

[31] Alharbi N Osman R Wismeijer D Effects of build direction on the mechanical properties of 3D-printed complete coverage interim dental restorations J Prosthet Dent 2016 115 06 760 767 26803175 10.1016/j.prosdent.2015.12.002

[32] Lauvahutanon S Takahashi H Shiozawa M Mechanical properties of composite resin blocks for CAD/CAM Dent Mater J 2014 33 05 705 710 25273052 10.4012/dmj.2014-208

[33] Kim K S Yoon T H Song K Y Ahn S G Comparison of mechanical properties in 4 indirect composite resin J Korean Acad Prosthodont 2007 45 21 33

[34] Revilla-León M Özcan M Additive manufacturing technologies used for processing polymers: current status and potential application in prosthetic dentistry J Prosthodont 2019 28 02 146 158 29682823 10.1111/jopr.12801

